# Yoga as adjunctive therapy in the treatment of people with anorexia nervosa: a Delphi study

**DOI:** 10.1186/s40337-021-00467-9

**Published:** 2021-09-08

**Authors:** Laura Rizzuto, Phillipa Hay, Melissa Noetel, Stephen Touyz

**Affiliations:** 1 School of Psychology, The University of Sydney, Sydney, Australia; 2 Translational Health Research Institute, School of Medicine, Western Sydney University, Greater Western Sydney, Australia; 3 University of Technology Sydney, Sydney, Australia; 4 InsideOut Institute University of Sydney, Sydney, Australia

**Keywords:** Anorexia Nervosa, Delphi, Yoga, Exercise

## Abstract

**Background:**

There is preliminary evidence to suggest that yoga can be beneficial in reducing anxiety, depression and general eating disorder symptoms in people with Anorexia Nervosa (AN). It is unclear whether the therapeutic benefits of yoga are supported or utilised in the treatment of AN amongst clinical experts. The present study aimed to explore and synthesise expert opinion on the use of yoga as an adjunctive therapy in the management of anxiety, depression and over-exercise in individuals with AN.

**Methods:**

A Delphi methodology was employed, with clinicians considered experts in the treatment of AN recruited internationally to form the panel (*n* = 18). The first iteration of questionnaires comprised of four open-ended questions concerning the experts’ understanding of the term yoga and opinions on its’ use in therapy generally and more specifically in the treatment of AN. Using content analysis, statements were derived from this data and included as Likert-based items in two subsequent rounds where panellists rated their level of agreement on each item. Seventeen out of 18 respondents completed all three iterations.

**Results:**

Consensus (level of agreement defined at ≥ 85%) was achieved for 36.47% of the items included in the second and third rounds. The panel reached consensus on items defining yoga and pertaining to its’ general benefits. The panel agreed that yoga is a adjunct therapy for various problems, consensus was not achieved on the specific use of yoga as an adjunct therapy in the treatment of comorbid anxiety, depression or trauma in patients with AN. Although the expert panel acknowledged a number of benefits for use of yoga in AN, they strongly endorsed that future research should evaluate the potential risks of using yoga as an embodied practice.

**Conclusions:**

It is possible that yoga could be considered for inclusion in future guidelines if supported by empirical research. We conclude that there seems to be enough consensus that such further scientific investigation is warranted.

**Plain English summary:**

This study aimed to explore expert opinion on the use of yoga as an adjunctive therapy in the management of anxiety, depression and over-exercise in individuals with Anorexia Nervosa (AN). Clinicians considered experts in the treatment of AN recruited internationally to form the panel (*n* = 18). Experts were asked about their understanding of the term yoga and their opinions on its’ use in therapy. The panel reached consensus on items defining yoga and pertaining to its’ general benefits. Although the panel agreed that yoga is a nice additional therapy for various problems, consensus was not achieved on the use of yoga as an additional therapy in the treatment of specific problems like anxiety, depression or trauma in people with AN. The expert panel acknowledged a number of benefits for use of yoga in AN. However the panel strongly considered that future research should evaluate the potential risks of using yoga as an embodied practice. The areas of collective agreement gained in the study can serve as preliminary guidelines for the use of yoga in AN whilst guiding future research directions.

## Background

Anxiety and depression are common comorbidities in individuals with Anorexia Nervosa (AN) [1–4]. While major depression has consistently been found in research to be the most commonly diagnosed comorbid disorder in individuals with AN [5, 6], anxiety has been associated with further disordered eating behaviours, a reduction in compliance with therapy and premature termination of treatment [4, 7–9]. As the primary focus of intervention in AN involves increasing weight from life-threateningly low levels, anxiety and depression are rarely the focus of therapeutic intervention [10] and tend to be managed with the use of atypical anti-psychotic medications with little evidence to support their efficacy [11, 12].

There is preliminary evidence to suggest that yoga can be beneficial in reducing levels of anxiety, depression and general eating disorder symptoms including self-objectification, body dissatisfaction, and drive for thinness in eating disordered populations [13–23]. Yoga is a practice traditionally rooted in Indian philosophy and spirituality comprising of ethical guidelines for living with an ultimate goal of attaining unification of mind, body and spirit [24]. Modern, predominantly Western conceptualisations of yoga mainly comprise of physical movement through postures (asana), breathing techniques (pranayama) and meditation (dhyana) [25, 26]. As such, the practice has become an increasingly popular means of promoting physical and mental wellbeing and is now recognised as a modality of complementary and integrative medicine [27]. Given that individuals with AN have been shown to control their symptoms of anxiety and depression with over-exercise [28], it has been suggested that the substitution of yoga over more strenuous forms of exercise may in fact address such concerns of compulsive exercise and the impact of weight loss in people with AN, whilst also reducing symptoms of anxiety and depression [29]. However, little evidence is available in support of yoga in the more severe presentations of eating disorders such as AN despite yoga being increasingly used in the treatment of eating disorders in both inpatient and outpatient settings [30–32].

It is unclear whether the therapeutic benefits of yoga are acknowledged, understood, supported, subsequently recommended or utilised in the treatment of AN amongst clinical experts. The present study aims to explore and synthesise expert opinion on the use of yoga as an adjunctive therapy in the management of anxiety, depression and over-exercise in individuals with AN and achieve a convergence of understanding. This will be used to inform further research directions in the field to ensure evidence continues to be clinically relevant and accessible.

## Methods

This study utilised the Delphi method, a technique widely employed in mental health research to establish the most reliable consensus of group opinion on a particular research area where knowledge is significantly lacking [33]. The process typically involves eliciting and refining the opinion of a diverse panel of experts on practice-related problems over a series of sequential questionnaires, interspersed with controlled feedback [34]. At each iteration stage, the panel’s collective response for each questionnaire item is summarized and presented to the panellists in later rounds where each expert is given the opportunity to rerate their items after considering the responses made by other panellists. The Delphi technique is commonly employed to address a lack of agreement or incomplete state of knowledge [35]. As such, the Delphi method was deemed an appropriate tool for the current study given the absence of evidence supporting the use of yoga as an adjunctive therapy in the treatment of AN.

### Participants and recruitment

Recruitment for participation commenced once ethics approval was gained by the University of Sydney Ethic Committee (2018/860). Participants were recruited using purposive sampling in order to identify experts in the field worldwide. Participants were identified by the authors as having highly specialized knowledge in the treatment of AN. To ensure that the panel for this Delphi study consisted of individuals with highly specialized knowledge of the target issue, the following inclusion criteria, as established by Noetel et al. [36] were used by the research team to identify and recruit a range of professionals deemed as experts according to the following standards: (I) established interest and expertise in the treatment of AN; and (II) distinguished contributor to the field of adolescent eating disorders as demonstrated by either: (i) current Fellow of the Academy for Eating Disorders (AED) status; (ii) membership of the Eating Disorders Research Society (EDRS); (iii) appointment as a Professor or Associate Professor in the field of AN; (iv) spent 10 years or more working in the field of AN; or (v) published peer-reviewed journal article(s) and/ or book(s) that are focused on AN. These membership lists along with key publications in the field of interest were used to identify potential panellists for the study. Based on the above inclusion criteria, a total list of 59 individuals were generated by the research team. This was in excess of the numbers we aimed to source (see following paragraph) and thus we stopped source sourcing potential participants at the point where we considered there were sufficient to conduct the study.

It is unclear within the literature the number of participants required to ensure a panel size is large enough to offer valid and reliable results when using a Delphi methodology [33, 35]. Guidelines suggest that numbers of participants will vary according to the scope of the problem and resources available, with little empirical evidence on the effect of the number of participants on the reliability or validity of consensus processes [34]. Whilst some studies have employed over 60 participants [37] others have involved a minimum of 15 [38]. Given the lack of empirical evidence, knowledge and understanding of the use of yoga in the treatment of AN, the current study aimed to recruit a sample of 20 participants. A total of 21 experts initially consenting to participation in the study, 18 of whom followed the first iteration of questions through to completion and 17 subsequently completed iterations two and three.

### Procedure

Three rounds of online questionnaires, developed using Qualtrics Research Suite survey software, were distributed to the final panel of 18 experts between February and August of 2019. Questionnaires were distributed in a synchronous format at the commencement of each round, and panellists were sent up to five email reminders for questionnaire completion to reduce attrition. Panellists were reminded of the anonymity of their specific responses at each iteration stage and given the option to be acknowledged in subsequent publications of this research.

### Defining consensus

**Fig. 1:**
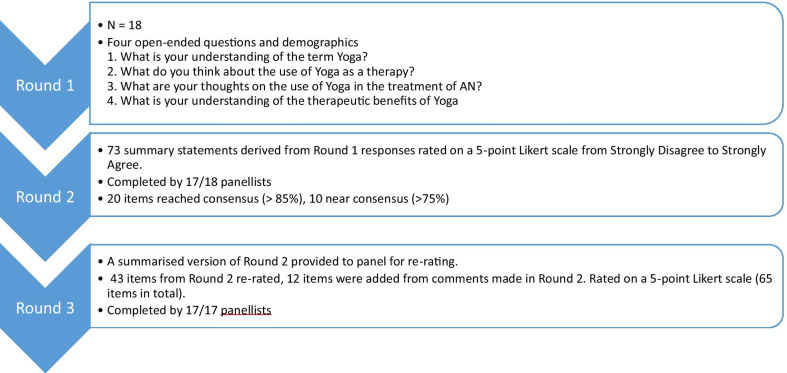
Flow diagram of response process

An established quantitative definition of consensus is lacking in the literature for Delphi studies [33] given the variability of factors such as research aim and panel size [34]. As such, a conservative consensus level of 85% level of agreement on item’s rating was set, consistent with previous studies [35, 36, 39]. Specifically, it was decided a priori that consensus was achieved when ≥ 85% of the panel or greater indicated agreement by selecting “strongly agree” or “somewhat agree” or indicated disagreement by selecting “strongly disagree” or “somewhat disagree” for an item. Near consensus was defined when ≥ 75% to < 85% of the panel showed either agreement or disagreement, and no consensus was confirmed when less than 75% of the panel demonstrated agreement or disagreement (see Fig. 1).

### Round 1

The first round of questionnaires included demographic items and specific questions to verify that the participant satisfied the inclusion criteria. Panellists were asked four open-ended questions (shown on Fig. 1) regarding their understanding of the term yoga and opinions on the use of yoga in therapy generally and more specifically in the treatment of AN.

### Round 2

Responses from Round 1 were analysed using content analysis to identify common themes in the data and corresponding summary statements which were subsequently used to develop a quantitative based questionnaire for Round 2 (73 items in total). Panellists were required to rate each item using a five-point Likert scale, with response options from “strongly disagree” to “strongly agree”. Taking into consideration comments made by the experts in round one, an additional item was added in Round 2 to determine whether panellists believed the benefits of yoga outweighed the risks in medically stable patients.

### Round 3

The collective responses for each item in Round 2 were analysed to establish the level of consensus reached amongst the panel. Items that reached the criteria for consensus were excluded (20 items), resulting in a subset of 53 items included in Round 2 for the panellists to rerate their level of agreement in Round 3 on the same five-point Likert scale. Each of these items included the participant’s previous response, as well as a bar graph representation of the percentage of responses for each level of agreement. This allowed participants to determine how their responses compared to the panel. Twelve new items were added to this round to account for comments made by the panellists in Round 2 increasing the item number to 65. A comments section was added for each item in this round so that participants were able to provide a rational for their final response and any additional comments they may have had regarding the topic of focus.

### Data analysis

A content analysis was used to analyse the qualitative data collected in Round 1 [40]. The panellist’s responses were compiled into common themes for each of the four focus questions, and similar responses merged into single statements for the panel to rate in subsequent rounds. For the purpose of reliability, statements included as items were reflective of the panellists’ own words. Data generated by respondents completed in Round 1 of the questionnaires was cross-coded by two of the researchers in the research team. Data collected from Rounds 2 and 3 were collated and analysed using Microsoft Excel. Frequency scores were calculated for each item to assess whether consensus had been achieved, and summary statistics including mean, standard deviation and mode were calculated to capture the collective judgements of the panel.

## Results

### Panellist response rate

A list of 59 individuals were generated by the research team, verified against the criteria for expert status, and invited to participate in the study. From the 59 individuals who were extended email invitations to participate, 5 (8.47%) declined due to work and family commitments, 33 did not respond to the invitation (55.9%), 21 consented to participate by clicking a link that redirected to the survey once the consent form was read (35.6%), with 18 completing past the demographics section and through the first iteration of questioning and the subsequent rounds (30.51%).

### Panellist characteristics

The panel of 18 experts consisted of 6 males (33.3%) and 12 females (66.67%), median age group range was 55–64 years (IQR 45–54 years to > 75 years) who had specialised in the area of eating disorders for a mean of 27.5 years (SD 5.34 years). The panel comprised of experts from diverse professional backgrounds (Table 1). All of the panellists worked predominantly in urban locations (city, large town), with none identified as working in rural or remote settings. At the time of study, 17 experts (94.44%) were engaged in clinical practice in a range of treatment settings. All panellists were active contributors to research in the field and had published peer reviewed journal articles and/or authored books, book chapters, or practice guidelines in Eating Disorders. Seventeen of the panellists (94.44%) had presented at eating disorder focused conferences. At the time of taking demographics, eight of the experts (44.44%) recommended yoga as an adjunctive therapy in the treatment of AN while 10 (55.56%) did not. Three of the experts (16.67%) were yoga practitioners ranging in practice duration from six months to 11 years. None of the experts who had a yoga practice were qualified teachers of yoga.

**Table 1:** Characteristics of study panellists (N = 18)

*Profession*	
Psychiatrist	8 (33.33%)
Psychologist	11 (45.83%)
Medical Doctor	1 (4.17%)
Researcher	2 (8.33%)
Lecturer	2 (8.33%)
*Geographical location of predominant workplace:*	
Australia	4 (23.53%)
USA	4 (23.53%)
UK	2 (11.76%)
New Zealand	1 (5.88%)
Canada	3 (17.65%)
Netherlands	1 (5.88%)
Israel	1 (5.88%)
Portugal	1 (5.88%)
*Highest level of education*	
PhD	8 (44.44%)
MD	2 (11.11%)
PhD/Masters combined program	2 (11.11%)
Masters & PhD completed separately	1 (5.56%)
Doctorate	3 (16.67%)
Masters	1 (5.56%)
Professional degree (i.e. 4 + 2 or 5 + 1)	1 (5.56%)
*How many adults with AN have you seen in your practice?*	
Fewer than 100	6 (33.33%)
100–500	4 (22.22%)
500–1000	2 (11.11%)
More than 1000	4 (22.22%)
Unknown	2 (11.11%)
*In what kind of treatment setting(s) do you currently work in, or have you previously spent considerable time working in?*	
Inpatient	12 (26.67%)
Residential	1 (2.22%)
Day patient	6 (13.33%)
Outpatient	13 (28.89%)
Private practice	11 (24.44%)
Other	2 (4.44%)
*When working with adults with AN, what is the primary focus of your role (e.g., providing psychological therapy, medical monitoring, research assessments, etc.)*	
Psychological therapy	12 (33.33%)
Research	8 (22.22%)
Clinical Assessment/diagnosis/recommendations	6 (16.67%)
Advise to families	1 (2.78%)
Consultation	2 (5.56%)
Medical monitoring	4 (11.11%)
Case management	1 (2.78%)
Psychiatric treatment	1 (2.78%)
Program management	1 (2.78)
*If you provide psychological therapy to adults with AN, what therapies do you use (all that apply)?*	
Cognitive Behavioural Therapy (CBT)	14 (19.71%)
Family-Based Therapy (FBT)	10 (14.08%)
Schema Therapy	4 (5.63%)
Acceptance and Commitment Therapy (ACT)	4 (5.63%)
Interpersonal Therapy (IPT)	4 (5.63%)
Dialectical Behaviour Therapy (DBT)	7 (9.86%)
Specialist Supportive Clinical Management (SSCM)	8 (11.27%)
Psychodynamic Psychotherapy	5 (7.04%)
Supportive Psychotherapy	9 (12.68%)
Eye Movement Desensitization & Reprocessing (EMDR)	2 (2.81%)
Narrative Therapy	1 (1.41%)
Emotional Recognition & Acceptance	1 (1.41%)
Psychological therapy currently not provided to adults with AN	2 (2.81%)
*Which one of the above therapies do you most often use or apply when treating adults with AN?*	
Cognitive Behavioural Therapy (CBT)	7 (38.89%)
Specialist Supportive Clinical Management (SSCM)	5 (27.78%)
Psychodynamic Psychotherapy	1 (5.56%)
Supportive Psychotherapy	2 (11.11%)
Other	2 (11.11%)
Psychological therapy currently not provided to adults with AN	1 (5.56%)
	M (SD)
On average, how many hours of your week are focused on individuals diagnosed with AN	14.4 (5.34)

### Final results

Consensus was achieved on 31 (36.47% agreement) of the total 85 items included in the second and third round, including 20 (27.4%) items (out of 73) reaching consensus in Round 2 and an additional 11 (16.92% out of 65 items) reaching consensus in Round 3. Of the remaining items, 14 (16.5%) were near consensus and 40 (47.06%) did not achieve consensus. The proportion of items that reached consensus in this study (i.e., 36.47%) was comparable with other research designs [41] in the eating disorder literature (e.g., 35.3%) and higher than those studies focused on the development of mental health management guidelines (e.g., 18.8% [42]; 22.8% [43].

#### Section A: defining yoga

Out of the fourteen items generated from round one related to a definition of what is yoga, 10 items reached consensus (60.7%), with two items near consensus (14.29%; see Table 2).

**Table 2:** Panelists’ responses on items for defining yoga, its general therapeutic benefits and use

Item	M	SD	Mode	% of panelists showing agreement	Consensus achieved
*Items for defining yoga*					
Yoga is a form of exercise focused on building strength, flexibility & balance	3.77	0.49	5	100%	Yes
Yoga involves body movement through postures	4.82	0.39	5	100%	Yes
Yoga involves focus on the breath	4.65	0.49	5	100%	Yes
Yoga encourages the individual to bring their attention to one’s own experience in their body including bodily sensations, breath and thoughts	4.65	0.61	5	94.12%	Yes
There are different forms of yoga, some more strenuous and vigorous (e.g. hot Yoga) and others gentler and calm, requiring less physical exertion	4.75	0.56	5	94.11%	Yes
Yoga involves a meditative component	4.29	0.85	5	88.24%	Yes
Yoga aims to integrate body and mind	4.47	0.72	5	88.23%	Yes
Yoga aims to enhance physical, mental and emotional wellbeing by reducing tension	4.41	0.71	5	88.23%	Yes
Yoga is an embodied mindfulness practice	4.41	0.71	5	88.23%	Yes
Yoga is a philosophy based on eastern traditions	4.41	0.71	5	88.23%	Yes
Yoga is a form of stretching	3.76	0.83	4	76.47%	Near
Yoga involves a spiritual component	4.06	0.75	4	76.47%	Near
*General therapeutic benefits of Yoga*					
Yoga has potential to improve quality of life	4.36	0.61	4	94.12%	Yes
Yoga helps develop physical strength and flexibility	4.59	0.62	5	94.12%	Yes
Yoga encourages connection to one’s body	4.59	0.62	5	94.12%	Yes
Yoga helps to develop strength without high impact exercise	4.41	0.71	5	88.24%	Yes
Yoga helps to develop mindfulness	4.06	0.56	4	88.24%	Yes
Yoga helps build emotional and physical wellbeing	4.24	0.66	4	88.23%	Yes
*General use of Yoga as an adjunct therapy*					
Yoga does not constitute a standalone therapy	5	0	5	100%	Yes
When well used, yoga can be a nice adjunct therapy for various problems, physically and emotionally	4.24	0.83	4	88.24%	Yes
Clinical judgement is required prior to recommending yoga as an adjunct to therapy	4.59	0.71	5	88.24%	Yes
Yoga is only useful in some, not all patients	4.47	0.87	5	88.24%	Yes
Any intervention, including yoga, depends on the client and needs to be tailored to their individual needs & preferences	4.18	1.01	4	88.24%	Yes
Yoga is a useful adjunct therapy for mood disorders	3.76	0.83	4	82.35%	Near

#### Section B: the general therapeutic benefits of yoga

A total of seven items were derived from the content analysis relating to the general therapeutic benefits of yoga. Out of these items, consensus was achieved on six (85.71%) with one item not meeting levels of consensus (14.29%) (see Table 2).

#### Section C: the general use of yoga as an adjunct therapy

Nine items were generated by the content analysis and one additional item included from Round 2 comments around the general use of yoga as an adjunct therapy. Of these ten items, five reached consensus (50%), one item near consensus (10%) with the remaining four items failing to reach consensus amongst panellists (40%) (see Table 2). Notably, all panellists agreed on the item ‘yoga does not constitute a standalone therapy’ whilst failing to reach consensus on the item ‘yoga should not be called therapy’ with 58.82% of panellists showing agreement (see Table 2).

#### Section D: yoga for the treatment of comorbidities found in AN

Three items were derived from the content analysis related to the treatment of comorbidities commonly found in patients with AN. It is notable that none of these items reached consensus, including ‘yoga can be beneficial for AN patients who have experienced trauma’ (41.18%), ‘yoga is beneficial in reducing anxiety in AN patients’ (64.71%) and ‘yoga is beneficial for AN patients experiencing depression’ (47.06%).

#### Section E: yoga as an adjunctive therapy in the treatment of AN

A total of twenty-one items derived from the content analysis alongside eight additional items accounting for comments made by the panel pertained to the use of yoga as an adjunctive in the treatment of AN. Five of these items reached consensus across rounds 2 and 3 (17.24%), with seven nearing consensus (24.14%), leaving 17 items (58.62%) without reaching agreement levels amongst panellists (see Table 3). It is notable that most of the items generated are cautionary statements about the use of yoga as an adjunct to the treatment of AN, for example, ‘AN patients will gravitate towards more strenuous forms of yoga (e.g. Hot yoga, power yoga) which are detrimental’.

**Table 3:** Panelists’ responses on items addressing the use of Yoga in the treatment of Anorexia Nervosa (AN)

Item	M	SD	Mode	% of panelists showing agreement	Consensus achieved
*Yoga as an adjunctive therapy in the treatment of AN*					
Yoga should only be recommended when the patient with AN is medically stable	4.59	0.80	5	94.12%	Yes
Yoga isn’t ‘girly’ and can also be undertaken by AN patients who are men or boys	4.65	0.70	5	88.24%	Yes
Positive responses regarding the use of yoga are not valid across the board for all AN patients, notably those in a very compromised physical condition	4.53	1.00	5	88.24%	Yes
AN patients will gravitate towards strenuous forms of yoga (e.g. hot yoga, power yoga) which are detrimental	3.76	0.75	4	88.24%	Yes
Yoga is beneficial for some AN patients where in others it can be harmful, particularly those who are medically compromised	4.41	0.71	5	88.24%	Yes
There is a risk that yoga is harmful because it could be used by AN patients to increase calorie expenditure	3.88	0.70	4	82.35%	Near
AN patients risk becoming perfectionistic and obsessional in their yoga practice	3.71	0.77	4	82.35%	Near
Not enough is known about yoga to comment on its use in patients with AN	4.06	0.83	4	82.35%	Near
Yoga can facilitate body awareness and acceptance in AN patients	3.88	0.60	4	76.47%	Near
Positive responses regarding Yoga are not valid across the board for all AN patients, notably those who oppose the idea of yoga	4.29	0.99	5	76.47%	Near
Questions about the use of Yoga in AN patients are problematic as they ask for an opinion when data is needed to support it	3.94	0.97	4	76.47%	Near
Clients need support with re-experiencing traumatic memories whilst doing yoga	4.06	0.75	4	76.47%	Near
*Therapeutic benefits of Yoga in the treatment of AN*					
Research is limited on the impact of yoga on AN	4.82	0.39	5	100%	Yes
It’s worth exploring in further research the therapeutic benefits of yoga in AN patients	4.59	0.62	5	94.12%	Yes
Offers a gentle reintroduction to physical activity for patients with AN	4.17	0.64	4	88.24%	Yes
There should be outcome measures that help the clinician know whether yoga is beneficial for an individual (e.g. body checking doesn’t increase, injury doesn’t occur, weight doesn’t go down)	4.47	0.72	5	88.24%	Yes
Research on the impact of yoga on AN would be beneficial	4.65	0.70	5	88.23%	Yes
Increased mindfulness in patients with AN	3.88	0.49	4	82.35%	Near
Focus on breath which is beneficial in AN patients	3.82	0.53	4	76.47%	Near
Increased experiential awareness of the body in patients with AN	3.88	0.60	4	76.47%	Near
Provides a break from the usual treatment routine for patients with AN	4.18	0.81	5	76.47%	Near

#### Section F: the therapeutic benefits of yoga in the treatment of AN

Of the 20 items derived from the content analysis and two added to account for Round 2 comments, five items reached consensus amongst panellists (22.73%) and a further 4 items were near consensus (18.18%). Most of the items generated failed to reach consensus (59.09%) with all panellists highlighting that ‘research is limited on the impact of yoga on AN’. Of note, four of the five items in this area that reached consensus pertain to the need for additional research and the use of outcome measures to monitor the potential risks associated with the use of yoga in patients with AN (eg. ‘There should be outcome measures that help the clinician know whether yoga is beneficial for an individual (body checking doesn’t increase, injury doesn’t occur, weight doesn’t go down)’; see Table 3).

## Discussion

The panellists reached a high level of consensus regarding a definition of yoga and agreed upon its’ general therapeutic benefits. All experts agreed that yoga is a form of exercise focused on strength, flexibility and balance that involves movement through postures and involves a focus on breath. Most acknowledged that yoga aims to integrate body and mind, is an embodied, mindful practice encouraging an individual to bring attention to their own experience in their body, including bodily sensations, breaths and thoughts that can enhance physical mental and emotional wellbeing. These descriptions of yoga are consistent with popular conceptualisations of the practice within Western culture [25, 26] that tend to place less emphasis on philosophical components of traditional practice. As such, it was unsurprising that, although some experts acknowledged the spiritual, ethical components of living a non-destructive life promoted by yoga philosophy [24, 44], the panel did not reach consensus on these items.

Although the panel reached criteria for consensus that ‘yoga is a nice adjunct therapy for various problems, physically and emotionally’, when presented with items generated pertaining to yoga’s usefulness in specific disorders, the opinions of experts were mixed. For example, panellists agreed on the usefulness of yoga as an adjunct therapy for mood disorders at a rate nearing consensus (82.35%), consistent with research in the field [45, 46]. Notably, none of the experts made comments or direct reference to bipolar disorder in the present study, possibly reflecting a dearth of research in this area [47]. Lack of research may also explain why comments made by experts around yoga for anxiety reduction were also not met with panel consensus, despite recent meta-analytical evidence demonstrating small short-term effects of yoga on anxiety compared to no treatment [48]. Additionally, whilst yoga was highlighted by some panellists as a useful adjunct therapy for trauma, when asked to rate and rerate collective opinion, the majority of experts selected ‘neither agree nor disagree’ as their response. As reflected by comments made by panellists, it is possible that some experts were not familiar with research conducted in the area and as such were tentative in providing an opinion [46, 48, 49].

Given the lack of consensus found regarding use of yoga in depression, anxiety and trauma, it was foreseeable that consensus would similarly not be achieved around the use of yoga to treat these symptoms when occurring concurrently in people with AN. Indeed, very few studies have examined the use of yoga specifically in patients diagnosed with AN [30, 31]. In a clinical RCT [30], Carei et al. found that a group of AN/BN patients with a comorbid DSM-IV diagnosis of psychotic disorder, conversion disorder, substance related disorder or/and an Axis II disorder demonstrated greater decreases in eating disorder symptoms (assessed with the Eating Disorder Examination) while Body Mass Index (BMI) was maintained compared to the control group (standard care only) when receiving the yoga intervention with standard care. However, both control and intervention groups demonstrated lower scores on anxiety and depression over time (using Beck Depression Inventory & State-Trait Anxiety Inventory) and thus reduced symptomology could be explained by the standard care each group received rather than the yoga intervention. Effect sizes were also small with low statistical power due to limited sample size, with no differentiation made between AN and Bulimia Nervosa (BN) patients. This suggests that, despite the availability of preliminary evidence regarding the effectiveness of yoga in alleviating anxiety and depressive symptoms, these findings are not yet supported in clinical samples with comorbid AN.

In contrast, it is noteworthy that the majority of items that reached consensus pertaining to the use of yoga as an adjunct therapy in AN populations consisted of cautionary statements about its use in clinical settings and thus should be carefully considered. Items included recommendations that yoga should only be used when the patient with AN is medically stable and that positive responses regarding the use of yoga are not valid across the board for all people with AN, notably those in a very compromised physical condition. Experts also expressed concern at a level reaching consensus that there is a risk of yoga being harmful because it could be used to increase calorie expenditure, that individual with AN may tend to be obsessional and perfectionistic in their practice and gravitate towards strenuous forms of yoga (e.g. hot yoga, power yoga) which are detrimental. Although some promising research has emerged on the use of yoga in eating disorder populations, none of these studies to date have assessed for potential risks of use of yoga outside of BMI. In Cramer et al.’s meta-analysis and systematic review of the safety of Yoga [50], they concluded that yoga appeared to be safe compared to treatment as usual except for in psychological/educational interventions where more yoga intervention-related adverse events and more non-serious adverse events occurred in the yoga group. Given the complexity of AN populations and their relationship with exercise, it is important that future research includes measures assessing potential risks for use of yoga amongst these patients. This was recommended at high levels of consensus in the present study, with experts expressing that there should be outcome measures that help the clinician know whether yoga is beneficial for the individual (e.g. body checking doesn’t increase, injury doesn’t occur, weight doesn’t go down).

It is also important to consider that unhealthy exercise has been found to be a major barrier to recovery from AN due to its association with a negative clinical profile and poor treatment outcomes [36, 51]. However, many authors have suggested that recovery from unhealthy exercise should move away from cessation and abstinence towards assisting the patient to establish a healthy, functional level of exercise, with accumulating evidence available in support of participation in structured exercise programs during treatment to assist in attenuating treatment-related symptoms, difficulties and distress [36, 52–54]. Although the panellists expressed caution regarding potential pitfalls for the use of yoga, they also endorsed at a level of consensus that yoga offers a gentle reintroduction to physical activity for patients with AN. Nevertheless, the importance of making carefully considered clinical judgements when recommending yoga was highlighted, that yoga is useful in some and not all patients, and that any intervention, including yoga, depends on the client and should be tailored to their individual needs and preferences. These comments are consistent with findings [36] where experts in the field of AN also advocated for the importance of clinical judgement in the reintroduction and promotion of healthy exercise.

Despite failing to reach consensus on the therapeutic benefits of yoga in people with AN, the panel strongly indicated that it’s worth exploring in further research. The panel delineated a number of potential benefits of yoga, with items around increased mindfulness, focus on breath, increased experiential awareness of the body and offering a break from the usual treatment routine nearing consensus. While some experts on the panel were familiar with research in the field and made comments about their personal experiences implementing yoga interventions with clients deemed to be suitable (e.g. “I have a direct experience of clients finding yoga to be a powerful intervention in supporting them to shift their relationship with AN through cultivating a more comprehension connection between their mind and body, in the practice of mindfulness to defuse from difficult thoughts and beliefs and also very importantly cultivation of self-compassion and kindness”), others commented that they were not familiar with the research data. Although it is reasonable for experts to be conservative around recommendations when there is limited research available, such comments suggest that clinicians in the field may not be aware of the current research on the use of yoga in clinical populations unless they have a personal or professional interest in its use.

### Strengths

To the authors’ knowledge, this is the first study to synthesise expert opinion on the use of yoga as an adjunct therapy in the management of anxiety, depression and overexercise in individuals with AN. A diverse panel of experts were recruited internationally for the study, and although panellist attrition is a common problem in Delphi studies [39], this study had strong retention rates across the three iterations (94.4% at round 1, 100% round 2 and 3).

### Limitations

The study limitations need to be acknowledged. Although this study had a strong retention rate across the three iterations (94.4%) the sample size was small. Additionally, the low response rate from individuals invited to participate may have resulted in sample bias. As such, those who participated in the study may have been more interested or held strong opinions of the research topic topic and/or were interested to participate for collegial reasons. Given that the findings are influenced by the composition of the expert panel, the results cannot necessarily be generalised to all clinicians involved in the treatment of AN. Further, as potentially more than half of the sample may not have had extensive knowledge or experience about yoga it is a limitation that those potentially in a position to integrate yoga into their practice appear not to be well-versed in the literature. Despite this, all individuals who participated in the study were considered experts in the field of AN. Given there is little research in the use of Yoga in people with AN, it is possible that those who did not respond to the study invitation preferred to withhold an opinion without a solid understanding of yoga or it’s therapeutic use. Experts contacted may also have refrained from response due to time constraints given the multiple contributions necessary across time points to generate reliable findings in a Delphi study. It is also acknowledged that findings may have differed with an approach to analysis such as that of Tierney and Fox [55] where responses are aggregated independent of those scored outside a range of consensus agreement and are thus less susceptible to a skew in the distribution of responses. Further, as the focus population for the current study was AN, these findings cannot be generalised to other eating disorder diagnoses. Additionally, the sex of the individual with AN was not specified for panellists and therefore conclusions cannot be made according to patient’s sex and as such future research may benefit from studying such variables. Future research may also benefit from examining whether treatment decisions differ depending on the clinical setting whilst also taking into consideration the opinions of other staff members such as nurses, GP’s, nutritionists and the patients themselves. It is also worth noting that experts unanimously agreed that yoga does not constitute a standalone therapy. As the study clearly focused on the use of yoga as an adjunctive therapy rather than a body-based activity that can be incorporated alongside treatment as usual, some participants commented that ‘yoga is not therapy’ and chose to ‘neither agree nor disagree’ with statements that described yoga as a therapeutic adjunct. This may have affected the findings, and as such, future research would benefit around the development of guidelines around whether yoga should be described as therapy or rather an activity that may hold therapeutic benefit for the patient.

## Conclusion and suggestions for future research

The current findings of the Delphi study provide insight into the expert opinion of the use of yoga as an adjunct therapy in people with AN. The areas of collective agreement gained in the study can serve as preliminary guidelines for the use of yoga in a population of AN patients whilst guiding future research directions. Although experts in the field acknowledge the general benefits of yoga, the findings strongly endorse that any future research should evaluate the potential risks of using yoga as an embodied practice alongside the benefits. It is important that such evaluations are made within high quality, controlled studies to further the reliability and validity of research in this area. It is possible that yoga could be considered for inclusion in future guidelines if supported by empirical research. We conclude that there seems to be enough consensus that such further scientific investigation is warranted.

## Data Availability

The datasets used and analysed during the current study are available from the corresponding author on reasonable request.

## Abbreviations

AN: Anorexia Nervosa
BN: Bulimia Nervosa
BMI: Body Mass Index
